# The Role of the Lawton Instrumental Activities of Daily Living (IADL) Scale in Predicting Adverse Events and Outcomes of R-CHOP Treatment in Elderly Patients with Diffuse Large B-Cell Lymphomas (DLBCLs) or Mantle Cell Lymphomas (MCLs): A Prospective Single-Center Study

**DOI:** 10.3390/cancers16244170

**Published:** 2024-12-14

**Authors:** Paula Jabłonowska-Babij, Magdalena Olszewska-Szopa, Stanisław Potoczek, Maciej Majcherek, Agnieszka Szeremet, Krzysztof Kujawa, Tomasz Wróbel, Anna Czyż

**Affiliations:** 1 Clinical Department of Hematology, Cell Therapies and Internal Medicine, Wroclaw Medical University, 50-367 Wrocław, Poland; magdalena.olszewska-szopa@umw.edu.pl (M.O.-S.); stanislaw.potoczek@umw.edu.pl (S.P.); maciej.majcherek@umw.edu.pl (M.M.); agnieszka.szeremet@umw.edu.pl (A.S.); tomasz.wrobel@umw.edu.pl (T.W.); a.czyz@umw.edu.pl (A.C.); 2 Statistical Analysis Centre, Wroclaw Medical University, 50-367 Wroclaw, Poland; krzysztof.kujawa@umw.edu.pl

**Keywords:** comprehensive geriatric assessment, elderly, R-CHOP, iADL, DLBCL, MCL

## Abstract

Background: The prognostic value of the comprehensive geriatric assessment (CGA) is recognized by many in hematology. However, there is no consensus on the utilization of alternative abbreviated methods to assess disabilities in elderly patients with B-cell non-Hodgkin’s lymphomas (B-NHLs). Aim: The aim of this study was to prospectively analyze the prognostic value of selected CGA tools in predicting adverse events (AEs) and outcomes of R-CHOP or R-CHOP-like treatment in elderly patients with diffuse large B-cell lymphomas (DLBCLs) or mantle cell lymphomas (MCLs). Methods: All patients who participated in this study underwent the Katz Index of Independence in Activities of Daily Living (ADL), the Lawton Instrumental Activities of Daily Living (iADL) scale, the Vulnerable Elders Survey-13 (VES-13), the Groningen Frailty Index (GFI), and the Mini Nutritional Assessment Short Form (MNA-SF) before starting anticancer treatment. Selected clinical predictors were also included in the study. Results: A total of 62 patients with newly diagnosed DLBCLs or MCLs, treated with R-CHOP in the Department of Hematology, Blood Neoplasm and Bone Marrow Transplantation of Wroclaw University Hospital between 1 July 2018, and 1 July 2020, were included in the study. The median age upon initiation of the treatment was 72 years (range: 61–68). Multinomial logistic regression and Cox proportional hazard regression analysis demonstrated that the iADL scale was significantly associated with response to treatment (OR = 1.21, 95% CI: 1.02–1.44, *p* = 0.03), was inversely related to non-hematological AEs (OR = 0.81, 95% CI: 0.71–0.92, *p* = 0.001), and was a statistically significant predictor of longer overall survival (OS) (HR = 0.83, 95% CI: 0.79–0.89, *p* < 0.001) and longer progression-free survival (PFS) (HR = 0.91, 95% CI: 0.83–0.99, *p* = 0.03). Conclusions: These results underscore the effectiveness of the iADL scale as a quick, easy-to-use, and universal CGA tool for evaluating crucial functional status before treatment in elderly hematological patients with DLBCLs or MCLs.

## Simple Summary

This prospective study evaluated the prognostic value of selected tools from the comprehensive geriatric assessment (CGA) in elderly patients with diffuse large B-cell lymphoma (DLBCL) or mantle cell lymphoma (MCL) treated with R-CHOP or R-CHOP-like regimens. A total of 62 patients were included in the analysis. The findings indicated that the Lawton Instrumental Activities of Daily Living (iADL) scale was a significant predictor of adverse events and treatment outcomes in this patient population.

## 1. Introduction

Non-Hodgkin’s lymphomas (NHLs) are the most common forms of hematological malignancies worldwide, representing approximately 3% of all cancer diagnoses and deaths [1]. Although the evidence indicates that the occurrence of cancer rises in the elderly, older patients are underestimated in clinical trials [2]. Therefore, in clinical practice, there is a lack of evidence-based medicine regarding the effectiveness and tolerance of anticancer treatments in the elderly population [3]. Furthermore, the many challenges encountered when treating elderly patients with hematological malignancies diminish their appeal as a study population for the purpose of discovering new therapies [4].

The health status of older patients exhibits significant heterogeneity, and the effectiveness of treatments differs based on frailty and physiological age rather than chronological age. It could be very difficult to customize treatments that maximize the effectiveness of treatment while minimizing therapy-related complications and toxicity [5]. Comprehensive geriatric assessment (CGA) is now the accepted “gold standard” of care for the elderly in hospitals. CGA includes assessments of functional status, physical health, mental function, and social–environmental situation and is used in elderly patients to determine eligibility for anticancer therapy [6].

The process of CGA is widely recognized as being highly time-consuming [2]. However, due to the ever-increasing number of elderly with hematological malignancies, research focused on finding quick and easy-to-use methods to assess the functional status of older patients before treatment becomes significant in predicting the adverse events (AEs), outcome of treatment, overall survival (OS), and progression-free survival (PFS) in this group [7].

The Instrumental Activities of Daily Living (iADL) scale is a widely utilized tool designed to assess an individual’s ability to perform essential tasks for independent living. These tasks encompass managing finances, handling transportation, shopping, preparing meals, performing housework, and managing medications. The iADL scale is administered through a questionnaire, which can be either self-reported by the patient or conducted by a healthcare professional, to evaluate the level of independence in performing these activities [8,9,10].

The iADL scale is distinguished by its structured approach and ease of administration. Each activity is scored according to the level of independence, ranging from complete independence to requiring full assistance. This straightforward scoring system enables quick and reliable assessments, making the iADL scale highly practical for use in clinical settings [8,9].

In the context of hematological malignancies, the iADL scale proves to be particularly invaluable. Patients with these diseases frequently undergo intensive treatments, such as chemotherapy and other aggressive therapies, which can significantly impact their functional status. Assessing a patient’s ability to perform instrumental activities of daily living enables healthcare providers to more accurately evaluate the patient’s overall functional capacity and resilience [11,12].

The aim of this study was to prospectively analyze the prognostic value of selected CGA tools in predicting AEs and outcomes of R-CHOP (rituximab, cyclophosphamide, doxorubicin, vincristine, and prednisone) or R-CHOP-like (R-CHOP with reduced anthracyclines) treatment in elderly patients with diffuse large B-cell lymphomas (DLBCLs) or mantle cell lymphomas (MCLs).

## 2. Materials and Methods

This study is a prospective analysis of all consecutive patients with newly diagnosed DLBCLs or MCLs and qualified for R-CHOP or R-CHOP-like treatment in the Department of Hematology, Blood Neoplasm and Bone Marrow Transplantation of Wroclaw University Hospital between 1 July 2018, and 1 July 2020, who met the inclusion criteria (see below) and signed the patient’s informed consent form (ICF).

Basic information was collected from the patient’s paper and electronic medical records, including gender, age, type of hematological disease, date of diagnosis, comorbidities, type of chemotherapy, and number of cycles. To thoroughly describe and analyze impact of other predictors in predicting AEs and outcomes, we also included the clinical stage, the Body Mass Index (BMI), the Eastern Cooperative Oncology Group (ECOG), the revised International Prognostic Index (R-IPI) or the MCL International Prognostic Index (MIPI), the Cumulative Illness Rating Scale (CIRS), and B-symptoms. The study also included selected local laboratory parameters, such as C-reactive protein (CRP), white blood cell count (WBC), hemoglobin (Hb), total cholesterol, lactate dehydrogenase (LDH), aspartate aminotransferase (AST), alanine aminotransferase (ALT), and albumin levels.

### 2.1. Inclusion and Exclusion Criteria

The study was assessed based on specific inclusion and exclusion criteria (see below).

Inclusion criteria were as follows: (i) subject is ≥60 years old at the time of providing ICF; (ii) subject must comprehend the information provided and willingly sign the ICF before any study-related assessments or procedures are performed; (iii) the subject has a confirmed diagnosis consistent with current guidelines for DLBCL or MCL; (iv) subject was never treated before due to DLBCL or MCL; (v) subject must receive treatment according to R-CHOP or R-CHOP-like treatment.

Exclusion criteria were as follows: (i) a subject with any major medical condition or psychiatric disorder that could interfere with their ability to participate in the study; (ii) the subject presents with any condition, including laboratory abnormalities, that would pose an unacceptable risk to their safety during participation in the study; (iii) the subject has any condition that could compromise the interpretation of study data; (iv) subject is undergoing another anticancer treatment.

### 2.2. Research Methods

The detailed clinical study protocol involved participants performing five tests:

1. The Katz Index of Independence in Activities of Daily Living (ADL);

2. The Lawton Instrumental Activities of Daily Living (iADL) scale;

3. The Vulnerable Elders Survey-13 (VES-13);

4. Groningen Frailty Index (GFI);

5. The Mini Nutritional Assessment Short Form (MNA-SF);

Tests were administered at first entry by a medical doctor or a clinical nurse specialist before the start of anticancer treatment.

ADL was discovered in 1959 by Katz et al. This tool has been created to evaluate the fundamental activities of daily living in elderly residing in the community or any care setting. The original iteration of the Katz Index included 8 elementary activities of daily living, but it was ultimately refined to include 6 basic activities: bathing, dressing, transferring, toileting, feeding, and continence. For the purpose of assessing this tool, a score of 1 is assigned if an older adult is able to perform an activity, and a score of 0 is assigned if the person is unable to perform it. The overall score ranges from 6 (maximum performance) to 0 (lack of performance). A score of 6 signifies full functionality, while a score of 4 suggests moderate impairment, and a score of 2 or lower indicates severe functional impairment [13].

The IADL scale evaluates 8 instrumental and functional activities: telephone use, shopping, meal preparation, housekeeping, laundry, transportation, medication management, and financial handling. The total score ranges from 0, indicating low functioning, to 8, reflecting high functioning and full independence. A patient scoring 6–7 points indicates mild dependence, needing minimal assistance; 4–5 points indicate moderate dependence, capable of performing some tasks independently; 2–3 points indicate significant dependence, needing help with most tasks; and 0–1 points indicate high dependence, requiring care. This scale may be completed in 10–15 min and provides an early indicator of functional deterioration or shows the necessity for additional evaluation [14,15].

VES-13 is a validated screening tool that includes 13 items, where the maximum score a patient can achieve is 10 points. It is used specifically in the elderly population to predict both functional decline and mortality. The VES-13 questionnaire requires patients to provide information about age, self-perceived health, and any functional impairments. Three or more points identify a geriatric patient and entail the need to perform CGA. As mentioned above, the questionnaire may be completed in 3–4 min [16,17].

GFI is a validated questionnaire that includes 15 elements. This tool examines the physical, cognitive, social, and psychological aspects and provides a score between zero and fifteen [18]. The questions are divided into four key geriatric domains: physical functioning (encompassing mobility, daily activities, vision, hearing, and weight loss), cognitive functioning, social functioning (with a focus on loneliness), and psychological functioning (including anxiety and depression). Higher scores signify greater frailty, with patients scoring 4 or more classified as frail [19].

MNA is a scientifically validated tool that was originally created to evaluate the nutritional status of older patients. This is primarily recommended for use in research environments. The tool consists of 18 items and evaluates four distinct areas: anthropometric measures (such as BMI, weight loss, and arm and calf circumferences); general assessment (addressing lifestyle, medication use, mobility, and indicators of depression or dementia); brief dietary review (including meal frequency, food and fluid intake, and feeding autonomy); and subjective assessment (focusing on self-perceived health and nutritional status) [20]. The MNA-SF is commonly utilized for rapid screening in clinical or community settings to swiftly identify individuals at risk of malnutrition. A score of 12–14 points indicates a normal nutritional status, 8–11 points indicate a risk of malnutrition, and 0–7 points indicate malnutrition [21]. Conversely, the MNA Full Form (MNA-FF) is used for a more thorough evaluation of nutritional status, frequently in research studies or detailed clinical assessments [22].

### 2.3. Statistical Analysis

Continuous variables are described as mean with standard deviation (SD) or median (Me) with minimum and maximum values. Categorical variables are presented as numbers of occurrence with percentages. A multinomial logistic regression analysis was used to assess the dependence treatment response of selected CGA tolls. The treatment response was categorized into 3 levels: improvement (complete response (CR) or partial response (PR)), no response (NR), and early death (ED). In multinomial logistic regressions, NR was chosen as the reference category for calculating odds ratios (ORs). The Box–Tidwell test is used to check whether the assumption of a linear relationship between log odds of the explained variable and continuous predictors is met.

The one-variable Cox proportional hazard regression analysis was performed to calculate hazard ratios (HRs), assessing the impact of individual CGA tools on OS and PFS. The assumptions of (1) proportional hazards and (2) linearity between the log hazard and predictors were evaluated using the Schoenfeld residuals test and Martingale residual plots, respectively. To summarize, the OR was used to evaluate risk factors related to the likelihood of developing the outcome of interest, regardless of the timing, while the HR was used to assess risk factors associated with the timing of outcome development. All ratios are presented with their respective 95% confidence intervals (CIs).

All statistical analyses were conducted using Statistica 13.3 (TIBCO Software Inc., Palo Alto, CA, USA (2017)).

### 2.4. Outcome

OS was calculated from the time of start of treatment to death (event) or the end of observation period (see below).

PFS was calculated from the time of start of treatment to disease progression or the end of observation period, 21 April 2024 (censored).

The final status of all patients was determined using both paper and electronic medical records. There were no instances of patients being lost to follow-up during the observation period.

### 2.5. Ethics

This study complies with the Declaration of Helsinki and was approved by the Wroclaw Medical University Ethics Committee (approval number: KB-414/2018; date of approval: 25 June 2018).

## 3. Results

### 3.1. Study Population

The study group consisted of 62 patients (41 men and 21 female) with a mean age of 72.77 ± 7.65 (SD) (range: 61–88) qualified for R-CHOP or R-CHOP-like treatment in the Clinical Department of Hematology, Cell Therapies and Internal Diseases of Wroclaw University Hospital between 1 July 2018, and 1 July 2020. Of these patients, 51 (82%) were diagnosed with DLBCL, and the remaining 11 (18%) had MCL. Prior to the start of anticancer treatment, each participant’s height and weight were measured, based on which the BMI was calculated. The mean value of BMI was 26.79 ± 4.69 (SD) (range: 19.02–40.44).

A detailed breakdown of these characteristics is presented in Table 1.

### 3.2. Selected CGA Tools vs. Response

In multinomial logistic regression analysis, the iADL scale was significantly associated with response to treatment (Table 2).

### 3.3. Selected CGA Tolls vs. OS

**Figure 1:**
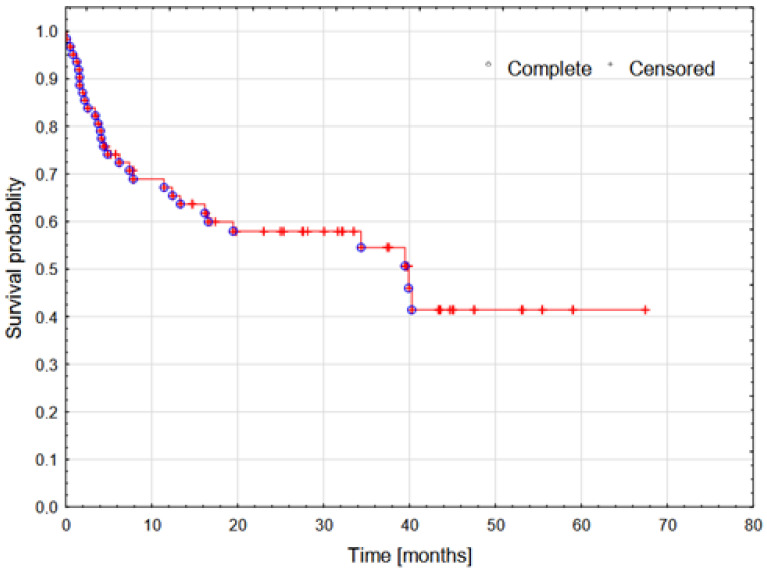
Kaplan–Meier survival curve for overall survival in the study group.

In Cox proportional hazard regression analysis, all CGA tools were statistically significant (Table 3). Of these tools, the ADL was the strongest predictor of OS due to its lowest HR, indicating a significant protective effect against mortality. The HRs < 1 (ADL, iADL, MNA-SF) suggest that better performance (higher result) is associated with lower mortality risk, whereas HRs > 1 (VES-13, GFI) suggest that worse performance (higher result) is associated with higher mortality risk. The probability of OS for all patients is presented using the Kaplan–Meier (KM) survival curve (Figure 1). A significant decline in survival is observed between months 40 and 50 of follow-up. After the 50-month mark, the curve stabilizes at approximately 40%.

### 3.4. Selected CGA Tools vs. PFS

**Figure 2:**
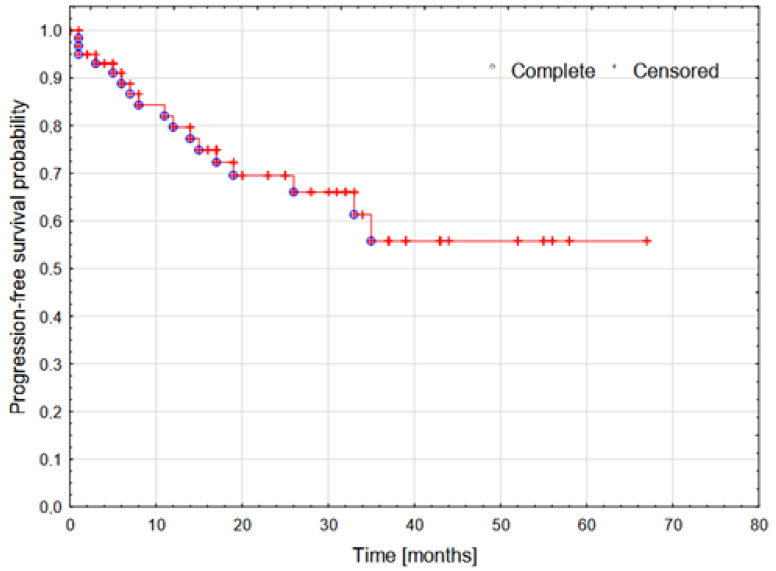
Kaplan–Meier survival curve for progression-free survival in the study group.

In Cox proportional hazard regression analysis, the ADL, iADL, VES-13, and GFI were significant predictors of PFS (Table 4). Of these tools, the ADL was the strongest predictor of PFS due to its lowest HR, indicating a significant protective effect against disease progression. The HRs < 1 (ADL, iADL) suggest that better performance (higher result) is associated with a lower risk of disease progression, whereas HRs > 1 (VES-13, GFI) suggest that worse performance (higher result) is associated with a higher risk of disease progression. The probability of PFS over time for all patients is presented using the KM survival curve (Figure 2). A significant decline in PFS probability is observed during the first 40 months of follow-up. Beyond the 40-month mark, the curve stabilizes at approx. 50%, suggesting that half of the patients did not experience disease progression or death in the subsequent observation period.

### 3.5. Selected CGA Tools vs. Non-Hematological AEs

In multinomial logistic regression analysis, all CGA tools were statistically significant (Table 5). Of these CGA tools, the iADL was the strongest predictor of the occurrence of AEs due to its lowest OR, indicating a significant protective effect. The ORs < 1 (iADL, MNA-SF) suggest that better performance (higher result) is associated with lower likelihood of AEs, whereas ORs > 1 (ADL, VES-13, GFI) suggest that a higher result is associated with higher likelihood of AEs.

### 3.6. Clinical Predictors vs. Response

In regression of response to treatment on clinical predictors, no significant predictor (*p* < 0.05) was found (Table 6).

### 3.7. Clinical Predictors vs. OS

In Cox proportional hazard regression analysis, ECOG, creatinine, and albumin levels were statistically significant (Table 7).

### 3.8. Clinical Predictors vs. PFS

In Cox proportional hazard regression analysis, ECOG was statistically significant (Table 8).

### 3.9. Clinical Predictors vs. Non-Hematological AEs

In multinomial logistic regression analysis, clinical stage of disease, BMI, ECOG, R-IPI and B-symptoms were statistically significant (Table 9).

## 4. Discussion

In this study, the prognostic value of selected CGA tools in predicting adverse events and outcomes of R-CHOP or R-CHOP-like treatment in elderly patients with DLBCLs or MCLs was prospectively analyzed. The standard assessment prior to the initiation of anticancer treatment was expanded to include five additional tests, identifying the medical, psychological, and functional limitations.

The latest international guidelines on qualifying patients with hematological malignancies for chemotherapy emphasize the importance of a multifaceted approach that considers both the biological characteristics of the tumor and the individual condition of the patient [23,24]. It is currently believed that chronological age is less significant than biological age and should not be the sole criterion for disqualifying a patient from anticancer treatment [25]. Properly qualifying elderly patients for chemotherapy is a significant step forward in optimizing care for this patient group. Early identification of patients more susceptible to complications related to cytostatic treatment will enable clinicians to appropriately intensify therapy and achieve the best possible treatment outcomes.

Currently, CGA which includes functional, psychological, and social evaluations serves as a valuable tool in the therapeutic decision-making process [25,26]. However, conducting all assessments requires significant time investment from the clinician, which can be very problematic due to the large number of patients [2]. Therefore, the search for and integration of simple-to-use and rapid tools for assessing the patient’s condition before the commencement of treatment into routine practice represents a significant research topic [27].

In this study, we suggest the significant and universal role of the iADL scale in predicting outcomes and occurrences of non-hematological AEs associated with R-CHOP or R-CHOP-like treatment in elderly patients with DLBCLs or MCLs de novo. Among the chosen screening scales from the CGA, only the iADL scale demonstrated a statistically significant association with response to treatment (OR = 1.21, 95% CI: 1.02–1.44, *p* = 0.03), longer OS (HR = 0.83 95% CI: 0.79–0.89, *p* < 0.001), longer PFS (HR = 0.91 95% CI: 0.83–0.99, *p* = 0.03), and the occurrence of non-hematological AEs (OR = 0.81 95% CI: 0.71–0.92, *p* = 0.001).

The obtained results demonstrate that a thorough assessment of a patient’s ability to perform daily activities is critical for evaluating their tolerance to anticancer therapy and predicting treatment outcomes. Conducting a simple iADL questionnaire can provide essential information for making individual therapeutic decisions.

Our findings align with recent studies that highlight the significant impact of the iADL score on the overall prognosis in elderly oncology patients. Doi A. et al. demonstrated that OS was significantly longer in patients with normal IADL compared to those with abnormal IADL (17.6 vs. 11.4 months, *p* = 0.049) [28]. Bila J. et al. showed that patients with iADL > 3 had significantly improved OS (log rank, 6.62; *p* < 0.001) [29]. Liu H. et al., using multivariable model analysis, identified two independent predictors of OS: one of them was the iADL result [30]. Wedding U. et al. established, in multivariate analysis, that impairment of IADL score (HR:4.3, 95% CI 1.7–10.5, *p* = 0.001) significantly predicted median survival [31].

Despite numerous findings similar to ours, we believe that further research should continue to explore and validate the use of CGA tools in diverse patient populations and treatment settings. This is important because there are publications in which the authors present different results. Goede V. et al. suggested that the iADL score had no statistically significant impact on the overall prognosis and that there is only a slight correlation between the iADL result and treatment toxicity [32]. Rollot-Trad F. et al. also obtained different results; in their multivariate analysis, only albuminemia was significant in the overall prognosis [33].

Interestingly, while ECOG and creatinine level were also significant predictors of OS in our study, no other predictors were found to be associated with treatment response in multivariate regression. This contrasts with a previous study conducted by Extermann et al. in which the authors’ analysis identified a broader range of predictors, including nutritional status, hemoglobin level, and comorbidities, as significant factors in elderly cancer patients’ outcomes [34]. A study by van Abbema et al. also presented severe comorbidities and nutritional status as significant predictors of treatment outcomes in elderly patients with cancer [35].

The discrepancy between our findings and those of other studies (both in relation to the iADL and clinical predictors) could be due to differences in study design, patient populations, or assessment methodologies.

To the best of our knowledge, this is the first prospective study to demonstrate the universal role of the iADL as a quick and easy-to-use tool that can be used in predicting treatment response, OS, PFS, and the occurrence of AEs in elderly patients with DLBCLs or MCLs. The obtained results seem to be of considerable significance given the steadily increasing number of hematological patients who will require individualized and optimized anticancer treatment.

## 5. Conclusions

In conclusion, our study highlights the crucial and universal role of the iADL scale in AEs and the outcomes of R-CHOP or R-CHOP-like treatment in elderly patients with DLBCLS and MCLs. The iADL scale’s effectiveness in prognostication and risk assessment supports its integration into routine clinical practice to tailor treatments and improve patient care.

## 6. Limitations

Our study has limitations. The first is the number of patients included. This limitation is due to strictly defined inclusion and exclusion criteria (26 patients did not meet the inclusion criteria). Secondly, certain information (such as specific laboratory results) is derived from patients’ medical records, which means that any unreported data have been excluded from our analysis. Nevertheless, thanks to thorough data collection and follow-up, the amount of missing data has been minimal. Third, participants may have inaccurately reported their functional status either by downplaying or exaggerating it, possibly due to a bias towards presenting themselves in a socially desirable way or because of cognitive impairment. Fourth, the sensitivity and reliability of our single-item questions may have been insufficient to fully capture the constructs of depression and loneliness. Finally, our study was conducted in a single center and may not generalize to other settings.

## 7. Future Research Directions

Based on the obtained results, which underscore the significant role of the iADL scale in predicting outcomes and AEs in elderly patients with DLBCLs or MCLs, a prospective clinical trial could be designed. It would be beneficial to conduct multicenter studies with a larger patient cohort to further validate these findings and enhance the generalizability of the results. In this study, patients would be stratified, according to their iADL scores, into two groups: (i) those receiving standard treatment (iADL score > 5 points); (ii) those receiving less intensive treatment (iADL score ≤ 5 points) [36].

A similar Phase II clinical study was conducted in Japan by Nakazato T. et al., utilizing the VES-13 questionnaire to personalize the type of therapy for patients with plasma cell myeloma (PCM). The results confirmed that tailoring treatment based on one questionnaire from CGA yields satisfactory outcomes (the study is registered as UMIN000011235) [16].

## Figures and Tables

**Table 1:** Patient characteristics and treatment details.

Characteristic	Number (%)
Total number of pts	62 (100)
Median age (years)	72
Gender	
Male	41 (66)
Female	21 (34)
Diagnose	
DLBCL	51 (82)
MCL	11 (18)
Clinical stage	
I–II	16 (26)
III–IV	46 (74)
R-IPI	
0 (very good)	0 (0)
1–2 (good)	12 (19)
3–5 (poor)	39 (63)
MIPI	
<5.7 (low)	1 (2)
5.7 to <6.2 (intermediate)	5 (8)
≥6.2 (high)	5 (8)
Constitutional symptoms	
Absent	27 (44)
Present	35 (56)
Type of treatment	
R-CHOP	45 (73)
R-CHOP-like	17 (27)
Treatment status	
Completed	46 (74)
Discontinued	16 (26)
Status for response	
Improvement (CR and PR)	46 (74)
NR	5 (8)
ED	11 (18)

Abbreviations: DLBCL—diffuse large B-cell lymphoma; MCL—mantle cell lymphoma; R-IPI—the revised International Prognostic Index; MIPI—the Mantle Cell Lymphoma International Prognostic Index; CR—complete response; PR—partial response; NR—no response; ED—early death.

**Table 2:** The regression of response to treatment on selected CGA tools.

Tool/ Predictor	Predictor’s Statistics						Model’s Statistics	
	Estimate	95% CIs	Wald Statistic	*p* Value	OR	95% CIs	Wald Statistic	*p* Value
ADL								
Improvement	0.44	(−0.06–−0.94)	2.98	0.084	1.56	(0.94–−2.57)	5.99	0.050
Early death	0.03	(−0.51–−0.57)	0.01	0.918	1.03	(0.60–−1.77)		
iADL								
Improvement	0.19	(0.02–−0.36)	4.64	0.031	1.21	(1.02–−1.44)	10.56	0.005
Early death	−0.01	(−0.20–−0.17)	0.02	0.902	0.99	(0.82–−1.19)		
VES-13								
Improvement	−0.18	(−0.50–−0.13)	1.26	0.261	0.83	(0.61–−1.14)	6.29	0.043
Early death	0.17	(−0.23–−0.57)	0.68	0.409	1.18	(0.79–−1.77)		
GFI								
Improvement	−0.26	(−0.59–0.06)	2.61	0.106	0.77	(0.56–1.06)	5.11	0.078
Early death	−0.02	(−0.37–0.32)	0.02	0.893	0.98	(0.69–1.38)		
MNA-SF								
Improvement	0.00	(−0.30–0.30)	0.00	0.990	1.00	(0.74–1.35)	2.69	0.260
Early death	−0.17	(−0.51–0.17)	1.00	0.320	0.84	(0.60–1.18)		

Abbreviations: ADL—Activities of Daily Living; iADL—the Lawton Instrumental Activities of Daily Living; VES-13—the Vulnerable Elders Survey-13; GFI—the Groningen Frailty Index; MNA-SF—the Mini Nutritional Assessment Short Form; CIs—confidence intervals; OR—odds ratio.

**Table 3:** The regression of OS on selected CGA tools.

Tool/ Predictor	Predictor’s Statistics				
	Estimate	95% CIs	*p* Value	HR	95% CIs
ADL	−0.48	(−0.69–−0.27)	<0.001	0.62	(0.5–0.76)
iADL	−0.18	(−0.25–−0.11)	<0.001	0.83	(0.79–0.89)
VES-13	0.44	(0.25–0.63)	<0.001	1.55	(1.28–1.88)
GFI	0.25	(0.14–0.37)	<0.001	1.29	(1.15–1.44)
MNA	−0.14	(−0.25–−0.03)	0.02	0.87	(0.78–0.97)

Abbreviations: ADL—Activities of Daily Living; iADL—the Lawton Instrumental Activities of Daily Living; VES-13—the Vulnerable Elders Survey-13; GFI—the Groningen Frailty Index; MNA—the Mini Nutritional Assessment; CIs—confidence intervals; HR—hazard ratio.

**Table 4:** The regression of PFS on selected CGA tools.

Tool/ Predictor	Predictor’s Statistics				
	Estimate	95% CIs	*p* Value	HR	95% CIs
ADL	−0.4	(−0.68–−0.11)	0.01	0.67	(0.5–0.9)
iADL	−0.1	(−0.18–−0.01)	0.03	0.91	(0.83–0.99)
VES-13	0.21	(0.03–0.39)	0.02	1.23	(1.03–1.48)
GFI	0.21	(0.07–0.35)	0.003	1.24	(1.07–1.43)
MNA	0.12	(−0.06–0.29)	0.19	1.13	(0.94–1.34)

Abbreviations: ADL—Activities of Daily Living; iADL—the Lawton Instrumental Activities of Daily Living; VES-13—the Vulnerable Elders Survey-13; GFI—the Groningen Frailty Index; MNA—the Mini Nutritional Assessment; CIs—confidence intervals; HR—hazard ratio.

**Table 5:** The regression of non-hematological AEs on selected CGA tools.

Tool/ Predictor	Predictor’s Statistics					
	Estimate	95% CIs	Wald Statistic	*p* Value	OR	95% CIs
ADL	0.39	(0.12–0.66)	8.09	0.014	1.48	(1.13–1.94)
iADL	−0.21	(−0.34–−0.09)	11.19	0.001	0.81	(0.71–0.92)
VES-13	0.28	(0.11–0.45)	9.97	0.002	1.32	(1.11–1.56)
GFI	0.39	(0.12–0.66)	0.09	0.004	1.48	(1.13–1.94)
MNA	−0.27	(−0.46–−0.09)	8.29	0.004	0.76	(0.63–0.92)

Abbreviations: ADL—Activities of Daily Living; iADL—the Lawton Instrumental Activities of Daily Living; VES-13—the Vulnerable Elders Survey-13; GFI—the Groningen Frailty Index; MNA—the Mini Nutritional Assessment; CIs—confidence intervals; OR—odds ratio.

**Table 6:** The regression of response to treatment on clinical predictors.

Clinical Predictor	Model’s Statistics	
	Wald Statistic	*p* Value
BMI	0.48	0.786
ECOG	3.92	0.141
MIPI	0.9	0.639
R-IPI	1	0.606
CIRS	0.05	0.974
CRP	4.26	0.119
WBC	0.16	0.923
Hb	2.07	0.355
Cholest	0.05	0.976
Creat	1.38	0.502
LDH	0.18	0.915
AST	0.59	0.745
ALT	0.05	0.975
Alb	2.96	0.227
B symptoms	0.34	0.845

Abbreviations: BMI—body mass index; ECOG—Eastern Cooperative Oncology Group; MIPI—Mantle Cell Lymphoma International Prognostic Index; R-IPI—the revised International Prognostic Index; CIRS—Cumulative Illness Rating Scale; CRP—C-reactive protein; WBC—white blood cell count; Hb—hemoglobin; Chol—total cholesterol; Creat—creatinine; LDH—lactate dehydrogenase; AST—aspartate aminotransferase; ALT—alanine transaminase; Alb—albumin.

**Table 7:** The regression of OS on clinical predictors.

Clinical Predictor	Predictor’s Statistics				
	Estimate	95% CIs	*p* Value	HR	95% CIs
Clinical stage	0.36	(−0.03–0.76)	0.072	2.07	(0.94–4.56)
BMI	−0.03	(−0.11–0.05)	0.447	0.97	(0.89–1.05)
ECOG	0.66	(0.26–1.06)	0.001	1.93	(1.3–2.88)
MIPI	1.96	(−0.56–4.51)	0.127	7.21	(0.57–91.03)
R-IPI	−0.19	(−0.51–0.13)	0.246	0.83	(0.6–1.14)
CIRS	0.005	(−0.09–0.1)	0.915	1.01	(0.91–1.11)
CRP	−0.01	(−0.02–0.0004)	0.058	0.99	(0.98–1)
WBC	−0.02	(−0.08–0.04)	0.455	0.98	(0.92–1.04)
Hb	−0.02	(−0.19–0.16)	0.828	0.98	(0.82–1.17)
Chol	0.002	(−0.007–0.01)	0.697	1	(0.99–1.01)
Creat	−2.08	(−4.1–−0.1)	0.04	0.12	(0.02–0.9)
LDH	0.0001	(0.0006–0.0008)	0.77	1	(0.1–1)
AST	−0.004	(−0.02–0.01)	0.63	0.1	(0.98–1.01)
ALT	−0.01	(−0.03–0.01)	0.442	0.1	(0.00–1.01)
Alb	−0.77	(−1.43–−0.1)	0.024	0.46	(0.24–0.91)
B symptoms	−0.26	(−0.65–0.12)	0.182	0.59	(0.27–1.28)

Abbreviations: BMI—body mass index; ECOG—Eastern Cooperative Oncology Group; MIPI—Mantle Cell Lymphoma International Prognostic Index; R-IPI—the revised International Prognostic Index; CIRS—Cumulative Illness Rating Scale; CRP—C-reactive protein; WBC— white blood cell count; Hb—hemoglobin; Chol—total cholesterol; Creat—creatinine; LDH—lactate dehydrogenase; AST—aspartate aminotransferase; ALT—alanine transaminase; Alb—albumin.

**Table 8:** The regression of PFS on clinical predictors.

Clinical Predictor	Predictor’s Statistics				
	Estimate	95% CIs	*p* Value	HR	95% CIs
Clinical stage	0.19	(−0.39–0.78)	0.529	1.45	(0.46–4.63)
BMI	0.02	(−0.08–0.12)	0.685	1.02	(0.92–1.13)
ECOG	0.52	(0.02–1.02)	0.042	1.68	(1.02–2.78)
MIPI	0.79	(−0.47–2.05)	0.221	2.2	(0.62–7.77)
R-IPI	0.46	(−0.15–1.08)	0.141	1.59	(0.86–2.95)
CIRS	−0.09	(−0.23–0.05)	0.187	0.91	(0.79–1.04)
CRP	−0.25	(−1.22–0.71)	0.609	0.78	(0.3–2.04)
WBC	0.01	(−0.05–0.06)	0.809	1.01	(0.95–1.07)
Hb	−0.16	(−0.4–0.09)	0.203	0.85	(0.67–1.09)
Chol	−0.01	(−0.02–0.008)	0.424	0.99	(0.98–1)
Creat	−1.12	(−3.05–0.8)	0.254	0.33	(0.05–2.24)
LDH	0.0001	(−0.0009–0.001)	0.841	1	(0.99–1)
AST	0.002	(−0.01–0.02)	0.739	1	(0.99–1.02)
ALT	0.0007	(−0.02–0.02)	0.949	1	(0.98–1.02)
Alb	−0.05	(−0.93–0.83)	0.907	0.95	(0.39–2.29)
B symptoms	−0.12	(−0.61–0.36)	0.614	0.78	(0.3–2.06)

Abbreviations: BMI—body mass index; ECOG—Eastern Cooperative Oncology Group; MIPI—Mantle Cell Lymphoma International Prognostic Index; R-IPI—the revised International Prognostic Index; CIRS—Cumulative Illness Rating Scale; CRP—C-reactive protein; WBC— white blood cell count; Hb—hemoglobin; Chol—total cholesterol; Creat—creatinine; LDH—lactate dehydrogenase; AST—aspartate aminotransferase; ALT—alanine transaminase; Alb—albumin.

**Table 9:** The regression of non-hematological AEs on clinical predictors.

Clinical Predictor	Predictor’s Statistics					
	Estimate	95% CIs	Wald Statistic	*p* Value	OR	95% CIs
Clinical stage	1.32	(0.11–2.54)	4.55	0.033	3.75	(1.11–12.65)
BMI	−0.15	(−0.27–−0.02)	5.08	0.024	0.87	(0.76–0.98)
ECOG	1.78	(0.79–2.78)	12.26	<0.001	5.95	(2.19–16.14)
MIPI	1.16	(−0.79–3.1)	1.36	0.244	3.17	(0.45–22.16)
R-IPI	0.75	(0.21–1.29)	7.32	0.007	2.11	(1.23–3.63)
CIRS	−0.02	(−0.15–0.11)	0.08	0.775	0.98	(0.86–1.12)
CRP	0.003	(−0.008–0.01)	0.27	0.607	1	(0.99–1.01)
WBC	0.03	(−0.05–0.11)	0.57	0.45	1.03	(0.95–1.11)
Hb	−0.21	(−0.46–0.04)	2.7	0.1	0.81	(0.63–1.04)
Chol	0.81	(−1.53–3.15)	0.46	0.5	2.25	(0.22–23.33)
Creat	0.02	(−1.29–1.33)	0.001	0.974	1.02	(0.28–3.79)
LDH	0.00	(−0.001–0.02)	0.54	0.463	1	(1–1)
AST	−0.01	(−0.03–0.02)	0.44	0.509	0.99	(0.97–1.02)
ALT	−0.02	(−0.05–0.01)	1.48	0.224	0.98	(0.96–1.01)
Alb	−0.83	(−1.84–0.19)	2.57	0.109	0.44	(0.16–1.2)
B symptoms	1.93	(0.81–3.05)	11.34	<0.001	6.86	(2.24–21.1)

Abbreviations: BMI—body mass index; ECOG—Eastern Cooperative Oncology Group; MIPI—Mantle Cell Lymphoma International Prognostic Index; R-IPI—the revised International Prognostic Index; CIRS—Cumulative Illness Rating Scale; CRP—C-reactive protein; WBC— white blood cell count; Hb—hemoglobin; Chol—total cholesterol; Creat—creatinine; LDH—lactate dehydrogenase; AST—aspartate aminotransferase; ALT—alanine transaminase; Alb—albumin.

## Data Availability

The raw data supporting the conclusions of this article will be made available by the authors on request.
